# Kinetics of Targeted Phage Rescue in a Mouse Model of Systemic* Escherichia coli* K1

**DOI:** 10.1155/2018/7569645

**Published:** 2018-07-11

**Authors:** György Schneider, Nikolett Szentes, Marianna Horváth, Ágnes Dorn, Alysia Cox, Gábor Nagy, Zsolt Doffkay, Gergely Maróti, Gábor Rákhely, Tamás Kovács

**Affiliations:** ^1^Department of Medical Microbiology and Immunology, University of Pécs, Medical School, Hungary; ^2^Department of Pharmacology and Pharmacotherapy, University of Pécs, Medical School, Hungary; ^3^Department of Biotechnology, Nanophagetherapy Center, Enviroinvest Corporation, Pécs, Hungary; ^4^Department of Biotechnology, University of Szeged, Hungary; ^5^Institute of Biochemistry, Biological Research Centre, Hungarian Academy of Sciences, Szeged, Hungary; ^6^Institute of Biophysics, Biological Research Center, Hungarian Academy of Sciences, Szeged, Hungary

## Abstract

*Escherichia (E.) coli* K1 strains remain common causative agents of neonatal sepsis and meningitis. We have isolated a lytic bacteriophage (ΦIK1) against* E. coli* strain IHE3034 and tested its specificity* in vitro*, as well as distribution and protective efficacy* in vivo*. The phage was shown to be specific to the K1 capsular polysaccharide. In the lethal murine model, a high level of protection was afforded by the phage with strict kinetics. A single dose of 1 x 10^8^ phage particles administered 10 and 60 minutes following the bacterial challenge elicited 100 % and 95 % survival, respectively. No mice could be rescued if phage administration occurred 3 hours postinfection. Tissue distribution surveys in the surviving mice revealed that the spleen was the primary organ in which accumulation of active ΦIK1 phages could be detected two weeks after phage administration. These results suggest that bacteriophages have potential as therapeutic agents in the control of systemic infections.

## 1. Introduction

The number of bacterial septicemia cases has been on the rise worldwide, affecting more than a million Americans [1], of whom between 28 and 50 % die [2].

After entering the body, survival and outcome of the hematogenous spread partially depends on the fitness and virulence factors of the invader. Certain factors enable bacteria to enter the central nervous system (CNS) and cause meningitis. The K1 serotype of* Escherichia (E.) coli* is the leading causative agent of Gram-negative bacterial meningitis with significant mortality and morbidity in newborns worldwide [3]. Its pathogenesis and pathophysiology have been investigated mostly using two* E. coli* K1 isolates originally isolated from the cerebrospinal fluid (CSF) of neonates with sepsis and meningitis [4]. Serotypes of these strains (RS218 and IHE3034) are identical, O18:K1:H7 [4]. The K1 capsule identical to the capsule of* Neisseria meningitides *B is thought to be one of the most important virulence factors as it supports survival in the host [5]. No vaccination is available for this capsule type because of the molecular mimicry between the serogroup B capsule and the tissue antigen of neuron cell adhesion molecule [6].

Capsule is not only a virulence associated factor, but a dominant outer surface cellular structure that is an ideal target for bacteriophages. These viruses can infect and multiply inside the bacterial host and, due to their specificity, they can be used to reduce target bacteria. Recently phage therapy has regained general interest as resistance to antibiotics has become a serious problem [7], and phage therapy has been found to be effective in treating many bacterial infections. Recent animal experiments have demonstrated the potential of phages as alternative therapeutic agents in various infection models ranging from topical wound and burn infections [8–10], pneumonia [11], and lung- [12] and intra-abdominal [13, 14] bacteremic infections.

In this work, we isolated a lytic bacteriophage specific for the K1 capsule of the newborn meningitis* E. coli* strain IHE3034 and demonstrated its therapeutic efficacy in a progressive intravenous mouse model. We demonstrated that intravenous administration of phages could effectively control progressive infections evoked by high bacterial cell number.

## 2. Materials and Methods

### 2.1. Bacterial Strains and Culture Conditions

The newborn meningitis* E. coli* (NMEC) strain IHE3034 (O18ac:K1:H7; phylogroup B2; sequence type ST95) was used throughout the study. It was originally isolated by 1976 in Finland [4] and kindly provided by the authors.

### 2.2. Bacteriophage Isolation

Bacteriophages against the NMEC strain IHE3034 were isolated from raw sewage water harvested from the Tettye Forrásház Treatment Plant located in Pécs-Pellérd, Hungary. Briefly, 5 ml sewage and 1 ml O/N culture of IHE3034 were added to 50 ml Luria-Bertani broth (LB) medium (Oxoid, USA) and incubated at 37°C and 150 rpm for 24 h. The following day, 1.5 ml of the infected LB-sewage mixture was harvested and centrifuged at 8,000 x* g *for 5 min to pellet cells and debris. From the supernatant, 980 *μ*l was transferred to a 1.5 ml reagent tube and 20 *μ*l of chloroform (Sigma, USA) was added and left at 4°C after shaking. After O/N incubation the sample was centrifuged (8,000 x* g *for 5 min) and a 100x serial dilution series (10^2^x, 10^4^x, 10^6^x, 10^8^x) was prepared for plaque forming unit (PFU) determination. Ten *μ*l from each dilution step was added to 4 ml of molten top agar (0.4% agar) tempered to 54°C and poured onto LB agar plates (1.5% agar) previously layered with lawns of logarithmic phase (OD_600_=0.8) culture of IHE3034. Plates were incubated O/N at 37°C. Phages were stored at 4°C, tested before use, and diluted accordingly.

### 2.3. Selection of Bacteriophages Specific for the K1 Capsule–K1 Mutant Construction

Phage plaques from the top agar plates were individually isolated and propagated in the presence of the wild-type IHE3034 NMEC strain. Tests of the phage suspensions were performed on the IHE3034 wild-type strain and effective phage plaques were picked up, propagated on IHE3034 K1, and counterselected on the lawn of its isogenic capsule mutant (ΔK1).

The capsule mutant was gained by using the Datsenko and Wanner method [15] based on the application of lambda Red recombinase expressing helper plasmid pKD46. Briefly, pKD46 was first electroporated into the wild-type IHE3034 and maintained at 30°C in the presence of Ampicillin (100*μ*g ml^−1^). PCRs were performed by using the primer pairs IHE_R2_pKDfw: 5′-TGGGTTTATTATGGGGGGAACACAACAAACTGCCAACATAATATATATTAAATTTCAAGTCAATATCTTTTGAATTTTAAGTGTAGGCTGGAGCTGCTTC, and IHE_R2-pKDrev: 5'-TTTATCTTAACAAAGAGGGGCAAGGTAAATTTTATAAAAATGTTACGGAAGCCATTGCTGATTACAAAAAAGACCTATAGCATATGAATATCCTCCTTAGTTCCTATTCC from the template plasmid pKD3 containing the* cat* cassette (encoding for the chloramphenicol resistance). The PCR product was electroporated into the freshly made competent cells of IHE3034 containing the L-arabinose (>10 mmol) induced lambda Red recombinase encoded on the pKD64 helper plasmid. Selection of the mutants was carried out in the presence of chloramphenicol. Elimination of Region2 of the capsule locus was tested by PCR by using the primers: kpsT_IHEell: 5′-CATGGCCCGTTGGATTGGC and KpsS_IHEell: 5′-CAGTACGGCGGGGATCTCT. Loss of the K1 capsule was phenotypically confirmed by latex agglutination (Thermo Fisher Scientific, USA) and designated as IHE3034 ΔK1.

Phages specific for K1 were purified three times on the IHE3034 strain by using the standard procedure described by Sambrook et al. [16].

### 2.4. Propagation and Purification of Phage Strains

High-titer phage stocks for* in vivo* experimentation and sequence analysis were propagated and amplified in the IHE3034 host bacterium strain by standard procedures [17]. For large-scale phage preparations for animal experiments the propagated phage and bacterium suspensions were centrifuged (2500 x* g*, 5 min) and the supernatants were filtered through a 0.22 *μ*m filter (Sarstedt, Germany), centrifuged (18000 x* g*, 30 min), and resuspended in 0.1M phosphate-buffered saline (PBS). Plaque forming units (PFUs) were determined and expressed in PFU ml^−1^, and stocks were stored at 4°C. These stocks were used for DNA isolation and subsequent sequencing.

### 2.5. Electron Microscopic Analysis

Morphology of phages was investigated by transmission electron microscopy as previously described [17]. Briefly, one drop of phage suspension was placed on copper grids with carbon-coated Formvar films and negatively stained with 2% ammonium molybdate (pH 6.8) for 1.5 min. The samples were examined in a Jeol 1010 transmission electron microscope (JEOL Inc. Peabody, MA USA) operating at 80 kV.

### 2.6. Pyrosequencing and Bioinformatics

Phage nucleic acid was isolated using the High Pure Viral Nucleic Acid Kit (Roche Applied Science, Mannheim, Germany), according to manufacturer's instructions. The complete genome of ΦIK1 was sequenced by the shotgun full-sequencing strategy using the GS Junior+platform (Roche Diagnostics GmbH, Germany). The mean coverage of ΦIK1 was 1380. The assembly of the sequence was performed with Geneious 8 software. Reads were mapped against the ΦIK1 genome sequence and the coverage was investigated for detection of direct repeats. Open reading frames (ORFs) were predicted using RAST [18].

### 2.7. *In Vitro* Activity Analysis of* E. coli* IHE3034 Treated with ΦIK1

*In vitro* activity was assessed to provide a quantitative analysis of the efficacy of the phage bactericidal activity in the presence of different phage-bacterium ratios. These tests were performed in a 96-well tissue culture plate. Briefly, O/N culture of IHE3034 was set to an OD_600_ of 1 (~2 x 10^8^ CFU ml^−1^) and the initial ΦIK1 bacteriophage suspension was set to ~2 x 10^8^ PFU ml^−1^).

The 96-well tissue culture plate was partitioned on four parallel sections. Each section contained 3 columns (1-3, 4-6, 7-9, 10-12) filled with 10^6^, 10^5^, 10^4^, and 10^3^ CFU ml^−1^ bacterial suspensions (180 *μ*l), respectively. Rows “A” and “H” contained sterile growth medium as a negative growth control (Figure 2). All other rows contained 20*μ*l of bacteriophage suspensions from the phage dilutions of 10^6^, 10^5^, 10^4^, 10^3^, and 10^2^. The final ΦIK1 phage concentrations were 10 times diluted (10^5^, 10^4^, 10^3^, 10^2^, and 10^1^ PFUs ml^−1^).

By using this checker board testing the following phage, bacterium multiplicity of infection (MOI) values were tested: 100:1; 10:1; 1:1; 1:10; 1:100; 1:1000; 1:10000; 1:100000. The assay was run for 24 h at 37°C. Bacterial growth was registered every 5 min by measuring the OD of each well with a multimode microplate reader (BioTek Synergy HT). The experiment was performed three times.

### 2.8. Mutation Rate Determination of the Receptor of ΦIK1

IHE3034 suspension (1 x 10^8^) was evenly spread on the surface of an LB agar plate and 10 *μ*l of the ΦIK1 suspension (1 x 10^8^ PFU mL^−1^) was added dropwise. The area of the cleared lytic zone was measured and emerged resistant colonies were determined. Determination was performed three times.

### 2.9. Lethal Dose of IHE3034 in the Intravenous Infection Mouse Model

For* in vivo* tests, 6-7-week-old female BALB/c (19-21 g) and NMRI (20-22 g) mice were purchased from Charles River (Germany). Animals were cared for in accordance with the guidelines of the European Federation for Laboratory Animal Science Associations (FELASA), and all procedures, care, and handling of the animals were approved by the Animal Welfare Committee of University of Pécs.

For infection, log-phase bacteria grown in LB broth were washed in PBS and the optical density was set to OD_600_=0.5 (~1 x 10^8^ CFU mL^−1^); OD_600_=1 (~2 x 10^8^ CFU mL^−1^); OD_600_=5 (~1 x 10^9^ CFU mL^−1^); and OD_600_=10 (~2 x 10^9^ CFU mL^−1^). Groups of 10 mice received intravenous doses of 100 *μ*l from the above suspensions, giving a final amount of ~1 x 10^7^; ~2 x 10^7^; ~1 x 10^8^; and ~2 x 10^8^ bacterial cells, respectively. Death and general conditions were recorded for the following 7 days and LD50 value for IHE3034 was determined on both mouse strains.

### 2.10. Therapeutic Efficacy of ΦIK1 in the Intravenous Mouse Model

This study was performed in strict accordance with the FELASA (Federation of European Laboratory Animal Science Associations) guidelines and recommendations. The animal experiments were approved by the ethical committee of the University of Pécs (Permit Number: BA02/2000-20/2011). Altogether 6 groups (6 mice per group) of 6-7-week-old female BALB/c mice (19-21 g) were used. Five groups were intravenously infected with 0.1 mL, OD_600_=5 (1 x 10^8^ CFU / mouse), IHE3034* E. coli* NMEC strain. One group served as a negative or bacteriophage control (GPC), receiving 0.1 ml bacteriophage suspension (1 x 10^8^ PFU ml^−1^). The bacterial control group (GBC) received only 0.1 ml OD_600_=5 bacteria (1 x 10^8^ CFU / mouse), while 0.1 ml bacteriophage suspensions (1 x 10^8^ PFU / mouse) were administered to all other groups 10 min (G10M), 1 h (G1H), 2 h (G2H), and 3 h (G3H) postinfection. General conditions and survival rates of the mice were permanently monitored for two weeks. Experiments were performed twice.

### 2.11. Assessing the Titers of ΦIK1 in Different Organs

Two weeks after phage therapy treatments, all mice were sacrificed and the concentration of active bacteriophages in the blood, brain, and spleen was determined. Organs were homogenized in 2.5 ml PBS with a blade stirrer for 5 seconds. Tissue suspensions were centrifuged (8,000 x* g *for 5 min) and chloroformed as described above. Ten *μ*l from these and the 100x diluted tissue suspensions were added to a bacterial lawn, incubated at 37°C, and PFUs were determined the following day. Detection limit of the test was 100 phage particles in 1 ml blood and 250 phage particles in the brain and spleen.

## 3. Results

### 3.1. Characterization of* E. coli* IHE3034 Bacteriophages Isolated from Sewage Water

**Figure 1:**
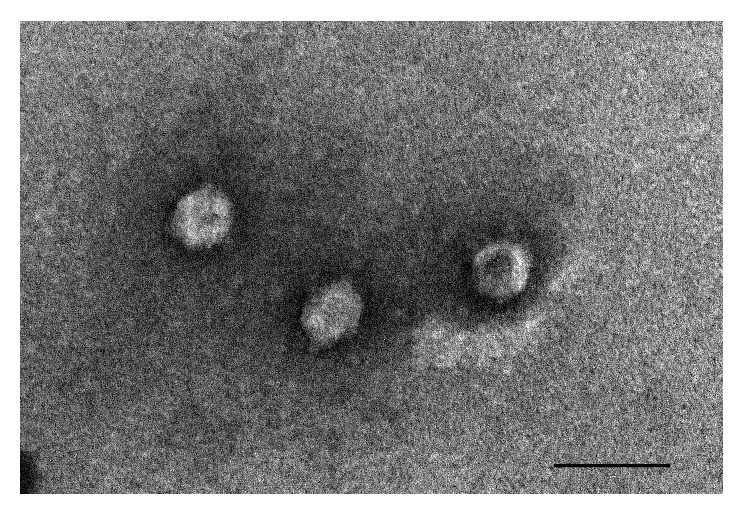
Morphology of ΦIK1 shows typical characteristics of* Podoviridae*. Bar represents 100nm.

Ten bacteriophages specific for the K1 capsule were purified from the local municipal sewage treatment facility of Pécs-Pellérd. Comparison of their DNA restriction profiles and recognition patterns on an* E. coli* strain collection has suggested that the 10 isolated phages were the same. One was chosen for further* in vitro* and* in vivo* studies and called ΦIK1. Transmission electron microscopy analysis of ΦIK1 indicates the typical characteristics of* Podoviridae* with a head diameter of ~80 nm (Figure 1.).

### 3.2. K1 Mutant Production from IHE3034 and Mutation Rate Determination

No clearing zone was observed on the capsule deficient isogenic mutant strain IHE3034 ΔK1 when 10*μ*l of ΦIK1 suspension was added to a bacteria-coated agar plate. However, it did cause a clear plaque in the presence of the wild-type IHE3034* E. coli* K1 strain.

The mutation rate of the receptor of ΦIK1 was 4.1 x 10^4^. The K1 agglutination assay revealed that 100% (34/34) of the tested colonies resistant to ΦIK1 lost the K1 capsule.

### 3.3. Sequence Analysis

Sequence analysis of ΦIK1 revealed that the genome size of ΦIK1 was 44246 bps with 94-96 % sequence similarity to 7 previously sequenced* E. coli* bacteriophages also belonging to* Podoviridae*. Altogether 58 ORFs of ΦIK1 were identified. The sequence of ΦIK1 was deposited in the Genbank under the accession number: KY435490.

### 3.4. *In Vitro* Activity Analysis

**Figure 2:**
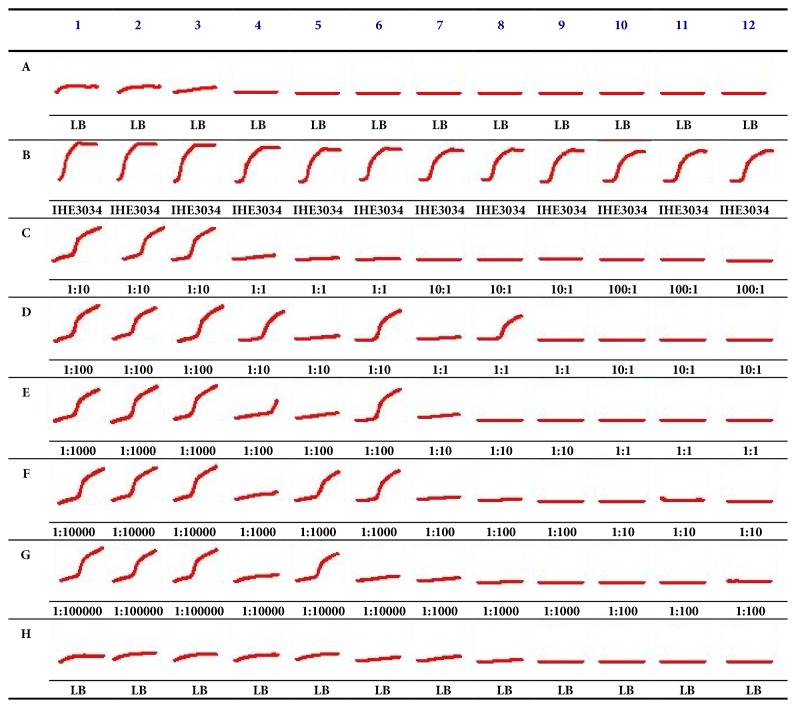
*In vitro* kinetics of the ΦIK1-IHE3034 interaction in different MOIs. Lines A (medium control), B (bacterium growth control), and H (medium control) are control fields. In rows C, D, E, F, and G the final concentration of phages are 10^5^, 10^4^, 10^3^, 10^2^, and 10^1^, respectively. In columns 1-3, 4-6, 7-9, and 10-12 bacterial end concentrations were 10^6^, 10^5^, 10^4^, and 10^3^ respectively. Accordingly ratios are presented PFU: CFU.

The 24 h* in vitro* time kill analysis revealed that ΦIK1 could hinder the proliferation of IHE3034. This inhibitory effect depended on the starting CFU of IHE3034 (Figure 2). If an MOI with a phage: bacterium ratio of 1:10 (10^5^:10^6^) was applied (see row C, columns 1-3) IHE3034 could overcome the attack of bacteriophage and by the end of the 24th hour the CFU number equaled the positive controls (only bacterium, no phage, row B). In contrast, ΦIK1 could successfully clear IHE3034 if at least a 1:1 bacteriophage: bacterium ratio was applied (MOIs: 10^4^:10^4^, D7-9; 10^3^:10^3^ E10-12).

At a bacterial concentration of 10^5^ CFU ml^−1^, the lytic effect of ΦIK1 was not significant as IHE3034 culture could avoid the inhibitory effect of ΦIK1 if the phage was diluted to 10^4^, 10^3^, 10^2^, and 10^1^ PFU ml^−1^ (D4-6, E4-6, F4-6, G4-6). This tendency changed if a 10^4^ CFU mL^−1^ bacterium suspension was tested in the presence of 10x, 100x, and 1000x less phage particles (MOI: 1:10; 1:100 and 1:1000; see wells E7-9, F7-9 and G7-9 respectively). In these cases the growth of bacteria was effectively hindered by ΦIK1. This was also observed if the bacterial cell number was further decreased to 10^3^ CFU ml^−1^.

This threshold at which IHE3034 could grow, at an inoculum of 10^5^ (Figure 2, column 4-6), is in accordance with the mutation rate of ΦIK1 receptor (~10^4^).

### 3.5. Intravenous Mouse Sepsis Model of IHE 3034

Two mouse strains were used to establish the systemic infection mouse model for IHE3034. BALB/c mice were more sensitive to IHE3034, as intravenous administration of 0.1 ml cell suspension (OD_600_=10) caused 100 % mortality in 24 h (SFigure 1.). Most of the mice survived if 0.1 ml suspensions of OD_600_=0.5 (1 x 10^7^ CFU) or OD_600_=1 (2 x 10^7^ CFU) bacterial cells were injected intravenously. Administration of 0.1 ml from OD_600_=5 (1 x 10^8^ CFUs / mouse) caused the death of all mice in 48 h.

In contrast, no complete mortality could be achieved in any of the NMRI mice groups treated with the above suspensions (SFigure 1.).

### 3.6. Phage Rescue of the IHE3034 Rapid Sepsis by ΦIK1

The bactericidal activity of ΦIK1 observed* in vitro* could be corroborated in the rapid sepsis mouse model. The restraining effect strongly depended on the time between infection and phage administration.

**Figure 3:**
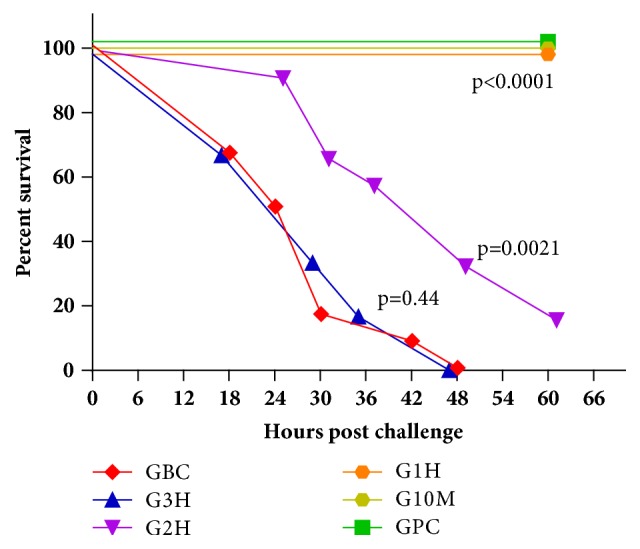
In the phage rescue experiment, survival of IHE3034 infected BALB/c mice strongly depended on the elapsed time between the injection of bacterium suspension (1 x 10^8^ CFU/mouse) and ΦIK1 (1 x 10^8^ PFU / mouse). Phage administration occurred 10 min (G10M), 1 h (G1H), 2 h (G2H), and 3 h (G3H) postinfection, while the IHE3034 and the ΦIK1 suspensions were used as positive (GBC) and negative (GPC) controls, respectively. The obtained survival curves were statistically compared by the logrank (Matel-Cox) test using the GraphPad Prism 6.0 software.

All 12 positive control mice (GBC) infected with 1 x 10^8^ CFU IHE3034 bacterium suspension died within 48 h (Figure 3). No effect was observed when phage suspension only (1 x 10^8^ PFU / mouse) was administered to healthy mice (group of phage control, GPC). Bacteriophages exerted a 100% curing effect if administered within 60 min of bacterial infection, saving the life of all infected mice.

Efficacy of phage treatment was drastically altered if administration of ΦIK1 followed bacterial challenge by 2 h (G2H). In this case, survival rate at 24 h was 91.6% (11/12), but it drastically decreased to 33.3% (4/12) by the 48th hour (Figure 3). Two mice survived from this group after the 60th hour and eventually recovered after 7-10 days. No rescue effect of ΦIK1 was detected, with a 0% survival rate at 48 h postinfection if phage was administered 3 h after bacterial infection.

### 3.7. Reisolation of Persisting ΦIK1 Phages from Different Organs

**Figure 4:**
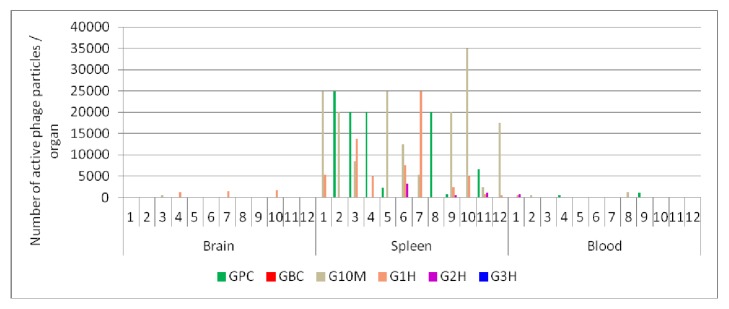
Detection of active phages in the surviving mice two weeks after bacterial challenge and phage rescue. Results are subdivided according to the organs (X axis). In each subsection (1-12) the summarized results of the GPC, G10M G1H, and G2H are presented as columns. G3H and GBC were not drafted as no mice survived in G3H, while no phage was administered to GBC (STable 1.).

Two weeks after bacterial challenge and subsequent phage administrations, surviving mice were sacrificed and active ΦIK1 phage particles were isolated from the blood, spleen, and brain (Figure 4,* x*-axis). The spleen was the only organ in which active phage particles could be detected in a significant amount, totalling <35000 PFU / spleen. The number of detectable phage particles was more limited in the brain (<1750 PFU / brain) and blood (<1250 *μ*l^−1^) (Figure 4). In the phage control group (GPC) no phage particles (0/12) were detected in the brain and a limited number of phages (<1000) were found in the blood of two sacrificed animals (2/12).

## 4. Discussion

Bacterial sepsis can progress if an aetiological agent enters the body and its hematogenous spread occurs. This condition strikes more than a million Americans [1] and has a high (28-50%) mortality [2]. Due to the emergence of multidrug resistance (MDR) among the aetiological agents it is increasingly obvious that new treatment options are needed [10]. The application of targeted bacteriophages could be a promising approach.

Counterselection of isolated lytic phages with the isogenic receptor mutant bacteria is an effective method to isolate targeted phages for a well-determined receptor. For this purpose, we have constructed and used the IHE3034 ΔK1 strain and isolated ΦIK1, targeting one of the most important virulence factors of the newborn meningitis-causing* E. coli* strain IHE3034 [19, 20]. We have chosen the K1 capsule as a target since it not only hinders opsonization [21], but also contributes to adhesion, promoting invasion through the blood-brain barrier (BBB) [19, 22].

Although the exact timing of the pathomechanism in IHE3034 in mice is not known, the 100% efficacy of the ΦIK1 treatment could be achieved if the phages were administered 1 h after bacterial challenge (G10M and G1H, Figure 3). There are several possible reasons for the inefficacy of phage rescue when performed after the first hour of infection (G2H and G3H), including the presence of phage resistant colonies in the inoculum. However their frequency was quite high (10^4^ ml^−1^) so resistant cells would have been incapable of evoking the pathogenic process. This is supported by the results of the* in vitro* experiments where ΦIK1 controlled IHE3034 at 1:1000 MOI (Figure 2), but only when the bacterial cell number was below 10^4^ CFU (the mutation rate).

Still, enough K1 expressing cells were in the blood of the experimental animals to take over a fatal pathogenic process. In our model system, IHE3034 could only trigger a rapidly escalating infection if the bacterium number was fairly high (1 x 10^8^ CFU / mouse). This is in accordance with other experimental setups performed with* Enterococcus faecium* as 10^9^ [13],* Staphylococcus aureus* as 10^8^ [23], and* Pseudomonas aeruginosa* as 10^7^ [14]. Compared to these experiments the 100% efficacy (G1H) of the phage rescue in relation of ΦIK1 and IHE3034 (1:1 MOI) is comparable to results of other studies, where 100% survival of the challenged mice were reported if ~1:3 (3x10^8^:1x10^9^), 10:1 (10^9^:10^8^) MOIs were applied and administration of phages occurred 45 min and 1 h after bacterial challenges [13, 23]. A more striking rescue effect was reported by Wang et al. [14] where the 1:100 MOI still had a 100% protective effect if the isolated* Pseudomonas*-specific phage was administered up to 1 h after the infection. This could be due to the fact that differences in the bacterium species used,* E. faecalis*,* S. aureus*,* P. aeruginosa*, and NM* E. coli*, possess different pathomechanisms. This strongly influences the availability of the infectious agent in the host.

Bacteria that are capable of intracellular survival, such as IHE3034 [20], can hide from bacteriophages and by this can assure their survival and lead to pathogenesis.* E. faecium* is able to survive and proliferate in murine macrophages [24] and there is also a mounting evidence that* S. aureus* has the potential to internalize and survive within host cells [25]. In contrast* P. aeruginosa* is thought to be an extracellular bacterium and therefore it is more vulnerable to bacteriophages. Intracellular bacteria are vulnerable until they reach, adhere to, and invade certain cells, protecting themselves from the extracellular phages. This may explain why phage rescue experiments were more effective with extracellular* P. aeruginosa*.

This process could be influenced by the tissue penetration potential of bacteriophages. The presence of ΦIK1 in the spleens of animals in the phage control group (GPC), and the surviving animals in groups G10M, G1H, and G2H, indicates that the phage could survive in the spleen for at least 2 weeks, until the mice were sacrificed.

Lack of ΦIK1 in the brains of the GPC group suggests that this phage cannot penetrate the BBB under normal conditions. However phage was detected in the brains of groups G10M and G1H (Figure 4.). We hypothesize that in group (G1H) ΦIK1 phages could also exert their lytic effects in the brain, contributing to rescue. At least two different mechanisms could be considered for this process. It is known that, upon inflammation, the BBB can become permeable, facilitating the transfer of macromolecules and viruses [26]. Our study contradicts this in the case of ΦIK1 as administration of 10^8^ heat-killed IHE3034 cells 1 h prior to the intravenous phage administration did not result in the detection of active phage particles in the brains of experimental animals when sacrifice occurred 1, 3, or 7 days after administration (unpublished data).

Based on this observation we postulate that the already phage-infected IHE3034 cells crossed the BBB into the brain tissue.

Phages are alternative therapeutics against multidrug resistant bacteria, but there are still questions about their practical use, especially in systemic infections. Besides the immunological aspects, a good understanding of phage-bacteria interactions and phage survival in the body is required. Our results demonstrate that, in accordance with previous findings, phages can not only combat bacteria already present in the circulatory system, but also survive in the spleen afterwards. Future work must be dedicated to those factors that influence this and possibly enhance the therapeutic potential of phages.

## Data Availability

The data used to support the findings of this study are available from the corresponding author upon request.

## Supplementary Materials

Supplementary Materials
** Supplementary figure **1**: concentration-dependent sensitivity of the BALB/c (A) and CD1 (B) mice for the IHE3034* E. coli* K1 meningitis strain on a time scale**. Ten-ten mice were intravenously injected with 100 *μ*L doses of OD_600_=0.5 (~1 x 10^8^ CFU mL^−1^); OD_600_=1 (~2 x 10^8^ CFU mL^−1^); OD_600_=5 (~1 x 10^9^ CFU mL^−1^); and OD_600_=10 (~2 x 10^9^ CFU mL^−1^) bacterial suspensions getting a final amount of ~1 x 10^7^; ~2 x 10^7^; ~1 x 10^8^; and ~2 x 10^8^ bacterial cells, respectively. Death rates are represented in both mice strains.** Supplementary Table **1**: detection of active phages particles in the different organs of the survived mice two weeks after bacterial challenge and phage rescue / administration** (Supplementary Table for Figure 4.).
