# Supplementary material for: Kinetics of Targeted Phage Rescue in a Mouse Model of Systemic Escherichia coli K1
Source: Biomed Res Int. 2018 Jul 11;2018:7569645. doi: 10.1155/2018/7569645 (PMC6076946; doi:10.1155/2018/7569645)
Supplement: Supplementary Materials — Supplementary figure 1: concentration-dependent sensitivity of the BALB/c (A) and CD1 (B) mice for the IHE3034 E. coli K1 meningitis strain on a time scale. Ten-ten mice were intravenously injected with 100 μL doses of OD600=0.5 (~1 x 10^8^ CFU mL−1); OD600=1 (~2 x 10^8^ CFU mL−1); OD600=5 (~1 x 10^9^ CFU mL−1); and OD600=10 (~2 x 10^9^ CFU mL−1) bacterial suspensions getting a final amount of ~1 x 10^7^; ~2 x 10^7^; ~1 x 10^8^; and ~2 x 10^8^ bacterial cells, respectively. Death rates are represented in both mice strains. Supplementary Table 1: detection of active phages particles in the different organs of the survived mice two weeks after bacterial challenge and phage rescue / administration (Supplementary Table for Figure 4.). [file 7569645.f1.docx]

**Supplementary figure 1. Concentration dependent sensitivity of the BALB/c (A) and CD1 (B) mice for the IHE3034 *E. coli* K1 meningitis strain on a time scale.**

Ten-ten mice were intravenously injected with 100 µL doses of OD_600_=0.5 (~1 x 10^8^ CFU mL^-1^); OD_600_=1 (~2 x 10^8^ CFU mL^-1^); OD_600_=5 (~1 x 10^9^ CFU mL^-1^); and OD_600_=10 (~2 x 10^9^ CFU mL^-1^) bacterial suspensions getting a final amount of ~1 x 10^7^; ~2 x 10^7^; ~1 x 10^8^; and ~2 x 10^8^ bacterial cells, respectively. Death rates are represented in both mice strains.

**Supplementary Table 1. Detection of active phages in the survived mice two weeks after bacterial challenge and phage rescue.** Supplementary Table for Figure 4.

|  |  |  |  |  |  | **Brain** |  |  |  |  |  |  |
| --- | --- | --- | --- | --- | --- | --- | --- | --- | --- | --- | --- | --- |
|  | 1 | 2 | 3 | 4 | 5 | 6 | 7 | 8 | 9 | 10 | 11 | 12 |
| GPC | 0 | 0 | 0 | 0 | 0 | 0 | 0 | 0 | 0 | 0 | 0 | 0 |
| GBC | - | - | - | - | - | - | - | - | - | - | - | - |
| G10M | 0 | 0 | 500 | 0 | 0 | 0 | 0 | 0 | 0 | 0 | 0 | 0 |
| G1H | 0 | 0 | 0 | 1250 | 0 | 0 | 1500 | 0 | 0 | 1750 | 0 | 0 |
| G2H | 0 | 0 | 0 | 0 | 0 | 0 | 0 | 0 | 0 | 0 | 0 | 0 |
| G3H | - | - | - | - | - | - | - | - | - | - | - | - |
|  |  |  |  |  |  |  |  |  |  |  |  |  |
|  |  |  |  |  |  | **Spleen** |  |  |  |  |  |  |
|  | 1 | 2 | 3 | 4 | 5 | 6 | 7 | 8 | 9 | 10 | 11 | 12 |
| GPC | 0 | 25000 | 20000 | 20000 | 2250 | 0 | 0 | 20000 | 750 | 0 | 6750 | 0 |
| GBC | - | - | - | - | - | - | - | - | - | - | - | - |
| G10M | 25000 | 20000 | 8500 | 0 | 25000 | 12500 | 5250 | 0 | 20000 | 35000 | 2500 | 17500 |
| G1H | 5250 | 0 | 13750 | 5000 | 0 | 7500 | 25000 | 0 | 2500 | 5000 | 750 | 500 |
| G2H | 0 | 2500 | 0 | 0 | 0 | 3250 | 0 | 0 | 500 | 0 | 1000 | 0 |
| G3H | - | - | - | - | - | - | - | - | - | - | - | - |
|  |  |  |  |  |  |  |  |  |  |  |  |  |
|  |  |  |  |  |  | **Blood** |  |  |  |  |  |  |
|  | 1 | 2 | 3 | 4 | 5 | 6 | 7 | 8 | 9 | 10 | 11 | 12 |
| GPC | 0 | 0 | 0 | 500 | 0 | 0 | 0 | 0 | 1000 | 0 | 0 | 0 |
| GBC | - | - | - | - | - | - | - | - | - | - | - | - |
| G10M | 0 | 500 | 0 | 0 | 0 | 0 | 0 | 1250 | 0 | 0 | 0 | 0 |
| G1H | 500 | 0 | 0 | 0 | 0 | 0 | 0 | 0 | 0 | 0 | 0 | 0 |
| G2H | 800 | 0 | 0 | 0 | 0 | 0 | 0 | 0 | 0 | 0 | 0 | 0 |
| G3H | - | - | - | - | - | - | - | - | - | - | - | - |
